# Thermodynamics Underpinning the Microbial Community‐Level Nitrogen Energy Metabolism

**DOI:** 10.1111/1462-2920.70055

**Published:** 2025-02-16

**Authors:** Mayumi Seto, Risa Sasaki, Hideshi Ooka, Ryuhei Nakamura

**Affiliations:** ^1^ Department of Chemistry, Biology, and Environmental Sciences Nara Women's University Nara Japan; ^2^ Biofunctional Catalyst Research Team RIKEN Center for Sustainable Resource Science Wako Saitama Japan; ^3^ Earth‐Life Science Institute (ELSI) Institute of Science Tokyo Tokyo Japan

**Keywords:** ecosystem functioning, energy metabolism, graph‐theoretical analysis, metabolic diversity, nitrogen cycle, redox chemistry, thermodynamics

## Abstract

Nitrogen compounds often serve as crucial electron donors and acceptors in microbial energy metabolism, playing a key role in biogeochemical cycles. The energetic favorability of nitrogen oxidation–reduction (redox) reactions, driven by the thermodynamic properties of these compounds, may have shaped the evolution of microbial energy metabolism, though the extent of their influence remains unclear. This study quantitatively evaluated the similarity between energetically superior nitrogen reactions, identified from 988 theoretically plausible reactions, and the nitrogen community‐level network, reconstructed as a combination of enzymatic reactions representing intracellular to interspecies‐level reaction interactions. Our analysis revealed significant link overlap rates between these networks. Notably, composite enzymatic reactions aligned more closely with energetically superior reactions than individual enzymatic reactions. These findings suggest that selective pressure from the energetic favorability of redox reactions can operate primarily at the species or community level, underscoring the critical role of thermodynamics in shaping microbial metabolic networks and ecosystem functioning.

## Graphical abstract

Energetically favourable nitrogen redox reactions align with community‐level networks, highlighting thermodynamic constraints shaping metabolism. Community‐level nitrogen energy metabolism shows stronger overlap with favourable reactions, suggesting selective pressure acts at the community level in microbial energy metabolism and ecosystem functions.

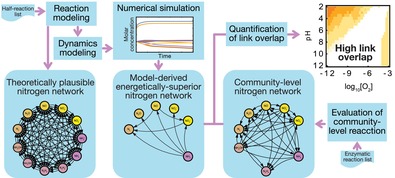

## 1 Introduction

Nitrogen compounds, with oxidation states ranging from −3 to +5, are key drivers of biogeochemical reactions on Earth (Falkowski et al. 2008; Stüeken et al. 2016). Microbes play a crucial role by catalysing essential reactions such as nitrogen fixation, nitrification, denitrification, anaerobic ammonia oxidation (ANAMMOX) and dissimilatory nitrate reduction to ammonium (DNRA) (Kuypers et al. 2018; Stein and Klotz 2016). These processes are integral to ecosystem productivity, greenhouse effects and water quality preservation. Consequently, microbial nitrogen metabolism has been critical for ecosystem functioning and will continue shaping biogeochemical cycles and informing environmental conservation strategies (Hutchins and Capone 2022; Liu et al. 2023; Nelson et al. 2016; Santoro 2010).

Beyond synthesising nitrogenous macromolecules, microbial nitrogen metabolism facilitates energy acquisition. Nitrogen compounds act as electron donors and acceptors, generating a proton motive force through the electron transport chain, ultimately synthesising adenosine triphosphate (ATP) (Burgin et al. 2011; Kuypers et al. 2018; Simon and Klotz 2013). However, the ability of energy metabolism to exploit a redox reaction involving electron transfer depends on enzyme functionality, as not all redox reactions can serve as energy sources. Given that the choice of energy‐sourcing reactions is directly linked to enhanced growth rates and fitness, the energetic favorability of redox reactions may act as a selective pressure, offering insights into the evolutionary constraints on microbial metabolic diversity (Crisp and Cook 2012; Großkopf and Soyer 2016; Wagner 2009).

The directionality and energetic favorability of a reaction can be quantitatively evaluated by the change in Gibbs energy during the reaction (∆_*r*_ *G*). Gibbs energy represents the thermodynamic potential inherent in the chemical compounds within a system. Enzymes do not directly alter the ∆_*r*_ *G* value but instead enhance reaction rates, thereby allowing microbes to extract energy at a biologically relevant timescale. Consequently, for an enzyme to exploit a specific energy‐sourcing reaction to evolve and enhance the host microorganism's fitness, the reaction must be intrinsically thermodynamically favourable for energy acquisition and supported by an adequate supply of reactants. This is exemplified by the discovery of ANAMMOX and complete ammonia oxidation (COMMAMOX), both of which were predicted purely through thermodynamic calculations as plausible energy‐sourcing reactions (Broda 1977; Costa et al. 2006; Daims et al. 2015; Kuenen 2008; Mulder et al. 1995). However, the extent to which thermodynamic properties have shaped the microbial nitrogen energy metabolism we observe today remains elusive and thus warrants further investigation.

Therefore, the central hypothesis of this study is that the redox reactions microbes have harnessed for energy acquisition—and for which they have evolved enzymatic functions—are fundamentally characterised by the thermodynamic potential of nitrogen compounds, which intrinsically constrains the energy yield of nitrogen‐based energy metabolism. The thermodynamic potential inherent in chemical compounds influences the energy available from reactions in two ways. First, the energy available from a reaction is determined by ∆_*r*_ *G*. Second, based on the second law of thermodynamics, the chemical composition of a system, which affects both ∆_*r*_ *G* and reaction rate, is influenced by the Gibbs energy driving force inherent in the thermodynamic stability of chemical compounds (Atkins and De Paula 2006; Kondepudi and Prigogine 2014). Although the second law of thermodynamics strictly applies to isolated systems with neither energy nor matter inflows, open systems such as ecosystems are also posited to move towards and remain in thermodynamically more stable states (Nielsen et al. 2020). Therefore, the reactions that microbes are likely to use as energy sources are those that exhibit high energy output, evaluated as the product of ∆_*r*_ *G* and reaction rate, under conditions characterised by the thermodynamic stability of nitrogen compounds.

To test this hypothesis, we quantitatively evaluated the similarity between energetically superior nitrogen reactions, identified from 988 theoretically plausible reactions based on geochemical and synthetic chemistry perspectives, and known microbial energy‐sourcing reactions. We first systematically analysed the energetic favorability of these reactions under simulated conditions with superior thermodynamic profiles. Subsequently, using graph‐theoretical approaches, we quantitatively assessed the similarity between the nitrogen network formed by these energetically superior reactions and the nitrogen community‐level network, reconstructed as a combination of enzymatic reactions representing intracellular to interspecies‐level reaction interactions. Our findings demonstrate that current microbial nitrogen energetic metabolism preferentially utilises energetically favourable nitrogen redox reactions, suggesting that thermodynamics has played a significant role in shaping microbial nitrogen energy metabolism within ecosystems.

## 2 Materials and Methods

All symbols and abbreviations used in this study are listed and defined in the Table S1.

### 2.1 Reactions Involving Nitrogen Compounds

Redox reactions are combinations of two half‐reactions, each explicitly involving the transfer of electrons in the reaction equation. For example,

(1) $${{\mathrm{NO}}_{3}}^{-}+2\;H^{+}+2\;e^{-}⇌{{\mathrm{NO}}_{2}}^{-}+H_{2}O$$

represents a half reaction where moving to the right corresponds to a reduction (electron acceptance), and moving to the left corresponds to an oxidation (electron donation). The electron transfer between this reaction and another half‐reaction, such as

(2) $$O_{2}+{\text{4H}}^{+}+{\text{4e}}^{-}⇌{\text{2H}}_{2}O$$

is achieved by balancing the stoichiometry of the electrons as follows:

(3) $${{\text{2NO}}_{3}}^{-}⇌{{\text{2NO}}_{2}}^{-}+O_{2}$$

In the dynamics model described later, we treat the leftward and rightward reactions of Equation (3) separately.

Although microbial energy‐sourcing reactions can involve organic nitrogen compounds such as urea, along with other elemental compounds like carbon, considering all possible combinations presents a significant challenge. Therefore, this study focuses exclusively on redox reactions that occur among 11 inorganic nitrogen compounds: NO_3_^−^, NO_2_^−^, NO, N_2_O, N_2_, NH_2_OH, NH_3_OH^+^, N_2_H_4_, N_2_H_5_^+^, NH_4_^+^, NH_3_, as well as O_2_, H^+^ and H_2_O. We comprehensively solved the combinatorial problem of half‐reactions, consisting of 37 half‐reactions of these nitrogen compounds and O_2_ + 4H^+^ + 4e^−^ ⇌ 2H_2_O, to derive redox reactions. The compiled half‐reactions, acid–base reactions and other reactions for the nitrogen compounds were sourced from geochemical and synthetic chemistry literature (see Supporting Information and Data S1). The algorithm for deriving redox reactions from half‐reactions was adapted from previous study (Seto and Kondoh 2023) and developed in the Wolfram Language (see Supporting Information and Data S1). Since the combination of half‐reactions can sometimes result in duplicates, removing redundancy produced 962 unique reactions. Subsequently, an additional 26 reactions, were added, bringing the total to 988 reactions.

### 2.2 Nitrogen Compounds Dynamics Model

The Gibbs energy change of a reaction represents its energetic favorability, while the energy profiles also influence reaction rates, driving the system towards a thermodynamically stable steady state (Atkins and De Paula 2006; Kondepudi and Prigogine 2014). Although natural environments do not always remain in a steady state, it is reasonable to consider that the energetic favorability of reactions at steady states, to which systems tend to converge over time, may have acted as a selective pressure on energy metabolism. Therefore, we describe the dynamics of nitrogen compounds under the influence of the Gibbs energy profiles and numerically solve for the concentrations of these compounds at the steady state. Given the focus of this study on exploring the extent to which thermodynamic properties of chemical systems influence microbial metabolism, the emergence of nitrogen energy metabolism‐based life and the resulting co‐evolutionary processes are not considered within the scope of this investigation.

The model represents an open homogeneous aquatic system, where the molar concentrations of 11 nitrogen compounds are expressed as the vector **M**(*t*) = ([NO_3_^−^], [NO_2_^−^], [NO], [N_2_O], [N_2_], [NH_2_OH], [NH_3_OH^+^], [N_2_H_4_], [N_2_H_5_^+^], [NH_4_^+^], [NH_3_]). The concentrations of dissolved oxygen and hydrogen ion are assumed to remain constant to exclusively focus on the dynamics of nitrogen species, which are the primary interest of this study. The dynamics of **M**(*t*) are governed by the following differential equations:

(4) $$\frac{dM\left(t\right)}{\mathrm{dt}}=I-DM\left(t\right)+Cr\left(t\right),$$

where **I** represents the influx rate vector, *D* the efflux rate constant, **C** the 11 × 988 stoichiometric coefficient matrix and **r**(*t*) the reaction rate vector:

(5) I=00⋮I,C=n1,1n2,1⋮n11,1n1,2n2,2⋮n11,2…………n1,987n2,987⋮n11,987n1,988n2,988⋮n11,988,rt=r1r2⋮r987r988

*n*_*k*,*i*_ represents the stoichiometric coefficient of the *k*th nitrogen compounds in the *i*th reaction (*rxn*_*i*_). All nitrogen compounds are exported from the system into the external environment in proportion to their molar concentrations. Ammonia was selected as the sole reactive and reduced nitrogen source due to the possibility that nitrogenase, which enables nitrogen fixation, existed before the development of nitrogen energy metabolism (Weiss et al. 2016), and that this flux may have been the largest, as it is on modern Earth (Gruber and Galloway 2008). In addition, ammonia may have likely played a predominant role as a nitrogen source on early Earth, particularly from deep‐sea hydrothermal vent emissions (Brandes et al. 1998).

### 2.3 Thermodynamic Weight on Reaction Rates

Because our objective is to identify energetically favourable reactions that are purely determined by the nitrogen thermodynamic potential, we place particular emphasis on understanding the effect of the Gibbs energy driving force on the development of microbial nitrogen energy metabolism by explicitly incorporating it into Equation (4). Consequently, unlike the Monod or Michaelis–Menten kinetics, which describe enzymatic reaction rates, we adopt rate expressions based on the law of mass action, the fundamental principle underpinning abiotic reaction processes:

(6) $$r_{i}=k_{i}\left(\Delta_{r}{G_{i}}^{{}^{\circ}}\right)\prod_{j=1}^{15}{\left[x_{j}\right]}^{n_{j,i}},$$

where *k*_*i*_(Δ_*r*_ *G*_*i*_°) represents the reaction rate constant for *rxn*_*i*_, which depends on the standard Gibbs energy change of *rxn*_*i*_. For simplicity, *k*_*i*_(Δ_*r*_ *G*_*i*_°) is denoted as *k*_*i*_ in the subsequent descriptions. [*x*_*j*_] denotes the molar concentration of the *j*th nitrogen compounds in the variable vector **MX**, and *n*_*j,i*_ is the stoichiometric number of the *j*th nitrogen compounds in *rxn*_*i*_. If we designate the leftward and rightward directions of the reaction in Equation (3) as *rxn*_1_ and *rxn*_2_, respectively, then the reaction rates are given by *r*_1_ = *k*_1_(Δ_*r*_ *G*_1_°) [NO_2_^−^]^2^[O_2_] and *r*_2_ = *k*_2_(Δ_*r*_ *G*_2_°) [NO_3_^−^]^2^, respectively.

The reaction rate constant is determined by both the magnitude of activation energy barrier and the Gibbs energy driving force (Ooka et al. 2023). To address the challenges associated with evaluating activation energies for each reaction, the model assumes equal activation energy across all reactions. This assumption is justified within the scope of our study, which focuses on the steady‐state dynamics because steady states are characterised by the Gibbs energy driving force, achieved through the dissipation of available energy within a system (Chapman et al. 2015; Nielsen et al. 2020). A reaction with ∆_*r*_ *G* < 0 proceeds relatively quickly compared with its reverse reaction, establishing *K*_eq_ = exp(−∆_*r*_ *G*°/*R* *T*) at equilibrium. Based on this relationship and incorporating the Brønsted (Bell)‐Evans‐Polanyi relationship, the effect of Gibbs energy driving forces on the rate constant *k*_*i*_ is defined by the following equation (Bligaard et al. 2004; Ooka et al. 2021, 2023; Ooka and Nakamura 2019):

(7) $$k_{i}=\left\{\begin{array}{cc} k_{\mathrm{def}}\exp\left(-\frac{p{∆}_{r}G_{i}^{\circ }}{\mathrm{RT}}\right) & \;\text{if}\;{∆}_{r}G_{i}^{\circ }<0\\ k_{\mathrm{def}}\exp\left(-\frac{\left(1-p\right){∆}_{r}G_{i}^{\circ }}{\mathrm{RT}}\right) & \text{otherwise} \end{array}\right.,$$

where *k*_def_ represents the reference reaction rate constant under the assumption that the activation energy is equal across all reactions. *p* (0 ≤ *p* ≤ 1) is a parameter controlling the thermodynamic weighting on the reaction rate, and the difference in reaction rates between the forward reactions with ∆_*r*_ *G*_*i*_° < 0 and their backward reactions becomes more pronounced as *p* increases. As *p* approaches 1, the reaction rate constants *k*_*i*_ for reactions with ∆_*r*_ *G*_*i*_° < 0 increase exponentially with smaller ∆_*r*_ *G*_*i*_° values, while *k*_*i*_ for reactions with ∆_*r*_ *G*_*i*_° > 0 converges to *k_def_*. Conversely, as *p* approaches 0, *k*_*i*_ for reactions with ∆_*r*_ *G*_*i*_° < 0 converge to *k_def_*, and *k*_*i*_ for reactions with ∆_*r*_ *G*_*i*_° > 0 converge to 0 as ∆_*r*_ *G*_*i*_° increases. By combining Equations (6) and (7), it becomes clear that the sign of ∆_*r*_ *G* determines the net reaction direction. Specifically, in Equation (3), if we designate *rxn*_1_ as the leftward reaction and *rxn*_2_ as the rightward reaction, and assume these two reactions alone determine the concentration of NO_3_^−^, $\frac{d\left[{\mathrm{NO}}_{3}^{-}\right]}{\mathrm{dt}}=-2k_{\mathrm{def}}\exp\left(-\frac{\left(1-p\right){∆}_{r}G_{2}^{\circ }}{\mathrm{RT}}\right){\left[{\mathrm{NO}}_{3}^{-}\right]}^{2}+2k_{\mathrm{def}}\exp\left(-\frac{p{∆}_{r}G_{1}^{\circ }}{\mathrm{RT}}\right){\left[{\mathrm{NO}}_{2}^{-}\right]}^{2}\left[O_{2}\right]$, where ∆_*r*_ *G*_2_° = −∆_*r*_ *G*_1_°. When ∆_*r*_ *G*_2_ < 0, $\frac{d\left[{\mathrm{NO}}_{3}^{-}\right]}{\mathrm{dt}}>0$, indicating that the leftward reaction dominates. Conversely, when ∆_*r*_ *G*_1_ < 0, the rightward reaction become dominant.

Although the simplicity of our model limits its precision for forecasting, this approach enables us to explore the energetic favorability of various redox reactions with minimal chemical parameter setup.

### 2.4 Numerical Simulation

The set of differential equations in Equation (4) was solved numerically until a steady state was reached using the NDSolve function in Mathematica 12. The efflux rate constant *D* and the absolute temperature *T* were fixed at 10^−3^ and 288.15 K, respectively. Initial values for all nitrogen species were set to 10^−5^ mol L^−1^. Oxygen concentration conditions (mol L^−1^) ranged from −12 ≤ log_10_ [O_2_] ≤ −3 in increments of 0.1, and pH conditions from 2 to 12 in increments of 0.5, totaling 966 conditions for the numerical differentiation calculation. The upper limit of oxygen concentration was set based on the saturation concentration of dissolved oxygen in Earth's surface waters (≈ 10 mg L^−1^ = 3.1 × 10^−4^ M). Calculations were performed under several conditions for the thermodynamic weighting coefficient *p*, the inflow rate of ammonia *I*, and the reference reaction rate constant *k*_def_, to examine the sensitivity of the system to each parameter.

## 3 Results

### 3.1 Thermodynamic Constraints on Steady‐State Nitrogen Compounds Composition

**FIGURE 1:**
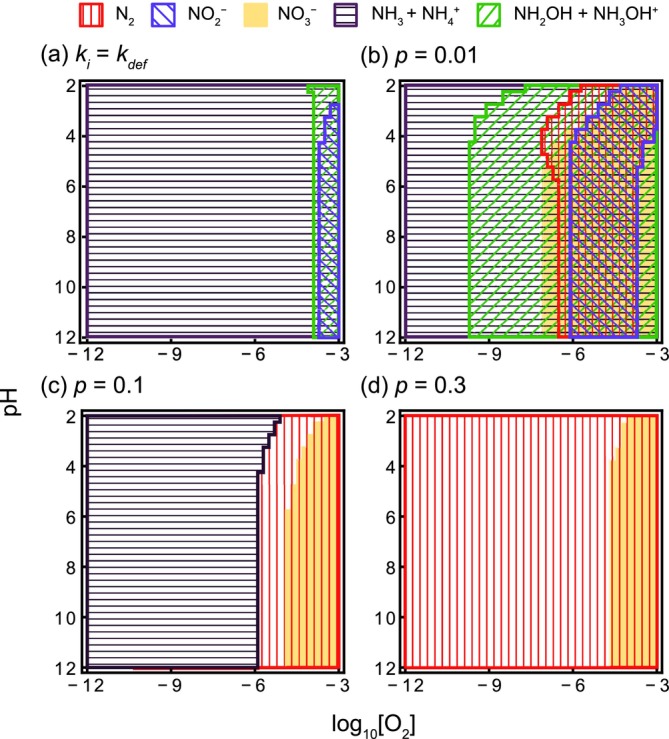
The combinations of major nitrogen species at each oxygen and hydrogen ion concentration in the steady state. The term ‘major nitrogen species’ refers to those that constitute more than 1% of the total concentration of the 11 nitrogen species. Each nitrogen species exists as a major nitrogen species in the respective patterns shown in the legend. (a) represents the scenario without considering thermodynamic weighting on the reaction rates: *kK*_*i*_ = *k*_def_. (b–d) correspond, respectively, to when *p* = 0.01, 0.1 and 0.3. *I* = 10^−5^, *D* = 10^−3^, *T* = 288 and *k*_def_ = 1.

The presence and magnitude of the Gibbs energy driving force, controlled by thermodynamic weighting parameter *p*, significantly influenced the steady‐state composition of nitrogen compounds by reflecting their reactivity (Figure 1). The absence of the Gibbs energy driving force (*k*_*i*_ = *k*_def_) or low *p* values underestimates the rates of energetically favourable reactions, which typically leads to the depletion of thermodynamically reactive compounds such as hydroxylammonium ion, resulting in the accumulation of this reduced nitrogen compound even in oxidative environments (Figure 1a,b). However, as *p* increases, reactions with small ∆_*r*_ *G* proceed relatively quickly, causing the prevalent nitrogen compounds under certain oxygen and pH levels to reflect those on present‐day Earth, where thermodynamically stable nitrogen in the form of N_2_ is abundant (Figure 1c,d and Figure S1). An increase in O_2_ levels enhanced the accumulation of oxidised nitrogen forms like NO_3_^−^, NO_2_^−^ and N_2_, while reducing forms like NH_2_OH, NH_3_OH^+^, NH_3_ and NH_4_^+^ decreased. Rising hydrogen ion levels favoured the formation of NH_3_OH^+^ and NH_4_^+^ due to protonation. The distribution pattern of nitrogen compound concentrations remained largely consistent across various NH_3_ supply levels (Figure S2).

### 3.2 Energetically Superior and Community‐Level Nitrogen Networks

**FIGURE 2:**
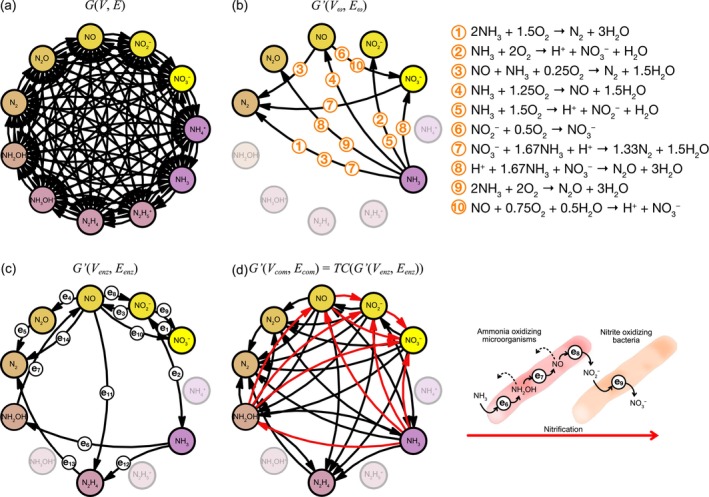
Nitrogen reactions as graphs. (a) The nitrogen reaction network comprising nitrogen species as nodes *V* with their transformations represented as directed links *E*, derived from the 988 reactions. (b) The energetically superior network formed from the top 10 reactions, ranked based on power *P_i_*, under steady‐state conditions with [O_2_] = 10^−5^ M, pH = 8, *p* = 0.3, *I* = 10^−5^, *D* = 10^−3^, *T* = 288 and *k*_def_ = 1. (c) The enzyme‐level network, formed from enzymatic reactions as reviewed by Kuypers et al. (2018), excluding nitrogen fixation. Each link *e*_*i*_ corresponds to a nitrogen transformation link for a specific enzymatic reaction. (d) The community‐level network depicted as the transitive closure of the enzyme‐level network. Red arrows show the transitive closure of the links from *e*_6_ to *e*_9_, which correspond to partial and complete nitrification.

We quantitatively assessed the similarity between model‐derived energetically superior reactions and actual microbial energy‐sourcing reactions using a graph‐based approach. We defined the graph *G*(*V*, *E*), which consists of 11 nitrogen compounds as nodes *V* and the directed links *E*, representing the nitrogen compounds transformation through 988 reactions (Figure 2a). The directed link set *E*_*i*_ for each reaction *rxn*_*i*_ includes all reactant–byproducts pairs, regardless of reaction selectivity or the preferential allocation of nitrogen atoms to a specific compound. For instance, the reaction NH_4_^+^ + NO_2_^−^ → N_2_ + 2H_2_O yields {(NH_4_^+^, N_2_), (NO_2_^−^, N_2_)}. The overall set *E* is the aggregation of directed links from all 988 reactions: $E=\cup_{i=1}^{988}E_{i}$. Each *rxn*_*i*_ is characterised by its steady‐state Gibbs energy change ∆_*r*_ *G*_*i*_, reaction rate *r*_*i*_ and power *P*_*i*_ = −∆_*r*_ *G*_*i*_ × *r*_*i*_, representing the energy output per unit time. Gibbs energy calculations were performed assuming activity coefficients to be 1, treating all nitrogen compounds as if they behave ideally. This assumption was made to account for the significant uncertainty in the ionic strength and the concentrations of coexisting ions in the pre‐nitrogen energy metabolism system. By assigning one of the rating values (∆_*r*_ *G*_*i*_, *r*_*i*_ and *P*_*i*_) to each *rxn*_*i*_ and selecting energetically superior reactions by sorting them in descending order based on those values, we delineate a subset of nitrogen compound nodes *V*_*ω*_ and directed links *E*_*ω*_, with the subscript *ω* representing *P*, *r* or − Δ_*r*_ *G* (Figure 2b). Depending on the analysis, this subset can be determined either by selecting the top 10 reactions or by choosing a fraction *θ* (e.g. *θ* = 0.9999) of the total energy output. Hereafter, the subgraph *G*'(*V*_*ω*_, *E*_*ω*_) will be referred to as ‘the energetically superior network’.

The fundamental units of the microbial nitrogen reactions are enzymatic reactions, with microbes executing sequential or partial nitrogen transformations through the expression of specific enzymes. Microbial enzymatic reactions specifically targeting energy acquisition from nitrogen compounds can also form a subgraph *G'*(*V*_enz_, *E*_enz_) (Figure 2c). The directed links *E*_enz_ = {*e*_1_, …, *e*_13_} represent the nitrogen transformations by 13 of enzymatic reactions (e.g. *e*_1_ = (NO_3_^−^, NO_2_^−^)), as reviewed by Kuypers et al. (2018), excluding nitrogen fixation. The specific combinations of links *e*_1_ through *e*_13_ correspond to DNRA (*e*_1_ and *e*_2_), denitrification (*e*_3_, *e*_4_ and *e*_5_), nitrification (*e*_6_, *e*_7_, *e*_8_ and *e*_9_) and ANAMMOX (*e*_3_, *e*_11_, *e*_12_ and *e*_13_). The role of ammonium (NH_4_^+^) in nitrification is still unclear (Wright and Lehtovirta‐Morley 2023); however, the enzymatic reaction catalysed by ammonia monooxygenase (e_6_ in Figure 2c) is assumed to specifically convert NH_3_ to NH_2_OH, rather than NH_4_^+^ to NH_2_OH (Jung et al. 2022; Suzuki et al. 1974). Hereafter, *G'*(*V*_enz_, *E*_enz_) will be referred to as ‘the enzyme‐level network’.

Each microbial species possesses a subset of these enzymes. For example, ammonia‐oxidising microorganisms (AOM) oxidise NH_3_ to NO_2_^−^ using enzymes denoted by *e*_6_, *e*_7_ and *e*_8_ (Caranto and Lancaster 2017). The cooperation between AOM and nitrite‐oxidising bacteria, which oxidise NO_2_^−^ to NO_3_^−^ using *e*_9_, completes the nitrification process, while intermediate products of AOM, NH_2_OH and NO, may leak out, enabling additional transitions from NH_3_ to NH_2_OH and from NH_3_ to NO. Such leakage of intermediate products has indeed been reported in denitrification and ammonification processes (Kits et al. 2019; Prosser et al. 2020; Sabba et al. 2017; Thakur and Medhi 2019; Yang et al. 2022). Consequently, the microbial nitrogen network, formed through the integration of multiple enzymes within the community‐level, should manifest as all possible combinations of enzymatic functions, represented by *G'*(*V*_com_, *E*_com_) (Figure 2d). *G'*(*V*_com_, *E*_com_) is the transitive closure of *G'*(*V*_enz_, *E*_enz_) and is identified using the TransitiveClosureGraph function in Mathematica 12. *G'*(*V*_com_, *E*_com_) will henceforth be referred to as ‘the community‐level network’.

When the set of links *E*_*ω*_ in Figure 2b matches the set *E*_com_ in Figure 2d, each link within *E*_*ω*_ is associated with either an enzymatic reaction or their composite reactions. For clarity, the numbered reactions in Figure 2b are referred to as “reaction X” throughout the remainder of this section. Reaction 1 can be considered as a composite reaction of nitrification and ANAMMOX, or nitrification and denitrification. Although the coexistence of these processes has been reported (Kuenen and Robertson 1994; Okabe et al. 2011; Poth and Focht 1985; Zhu et al. 2013), recent evidence suggests the presence of microbial candidates that undergo these reactions at the individual level, termed as direct ammonia oxidation with a potential new enzyme (DIRAMMOX) (Ouyang et al. 2021; Pan and Liu 2023; Wu et al. 2021). Reaction 2 is identified as complete nitrification, while Reactions (4), (5), (6) and 10 are its partial reactions. Reaction 3 resembles ANAMMOX but involves the utilisation of oxygen, which can be considered as ANAMMOX occurring with partial nitrification of NO to NO_2_^−^ by using O_2_ as an electron acceptor. Reactions 7 and 8 can be considered ANAMMOX performed after reducing NO_3_^−^ to NO_2_^−^ using the reducing power of NH_3_. Reactions 8 and 9, with N_2_O as the final product, are associated with partial denitrification.

### 3.3 The Overlap Rate Between the Model‐Derived and Microbial Community‐Level Nitrogen Networks

At each given O_2_ and pH conditions, we assessed the similarity between the energetically superior network and both the enzyme‐level and community‐level networks by comparing the edge overlaps:

(8) $$D_{\mathrm{enz},\omega }=\frac{\left|E_{\mathrm{enz}}\cap E_{\omega }\right|}{\left|E_{\omega }\right|}$$

(9) $$D_{\mathrm{com},\omega }=\frac{\left|E_{\mathrm{com}}\cap E_{\omega }\right|}{\left|E_{\omega }\right|},$$

where |*E*| represents the number of links within *E*. For example, in Figure 2b, the links derived from the top 10 reactions ranked by power *P* (*|E*_*P*_ *|* = 8) completely match the community‐level links (|*E*_com_ ∩ *E*_*P*_| = 8), resulting in *D*_com,*p*_ = 1. A higher overlap rate suggests that energetically superior reactions are selected as energy‐sourcing reactions, reinforcing the hypothesis that the energetic favorability of redox reactions has acted as a selective pressure on microbial energy metabolism.

**FIGURE 3:**
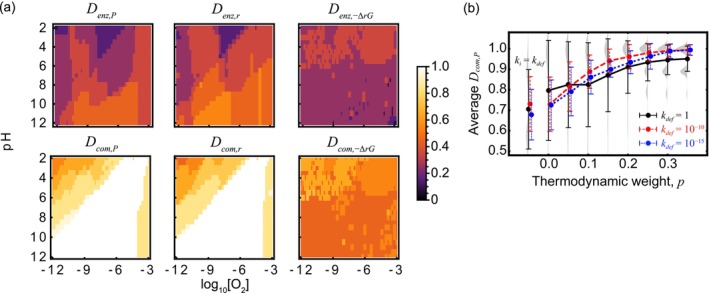
Edge overlap rates between the enzyme‐level or community‐level network and the energetically superior network, constructed from the top 10 reactions ranked by power (*P*), rate (*r*) and negative Gibbs energy of the reaction (−∆_*r*_ *G*). We denote these overlaps as *D*_enz,*ω*_ and *D*_com,*ω*_, where ω = *P*, *r* or − ∆_*r*_ *G*. (a) Edge overlap rates for each pH and oxygen concentration, with the top and bottom rows showing *D*_enz,*ω*_ and *D*_com,ω_, respectively. The columns from left to right correspond to energy rating factors by *P*, *r* and − ∆_*r*_ *G*, respectively. *k*_def_ = 1 and *p* = 0.2. (b) Average edge overlap rate *D*_com,*P*_ in relation to the reference reaction rate constant (*k*_def_) and thermodynamic weight (*p*), and in the absence of thermodynamic weighting (*k*_*i*_ = *k*_def_). The average edge overlap is calculated as the mean of edge overlaps under various oxygen concentrations and pH conditions. The distribution of *D*_com,*P*_ shown in grey corresponds to *k*_def_ = 1. *I* = 10^−5^, *D* = 10^−3^ and *T* = 288.

Figure 3 shows the similarity between the enzyme‐level or community‐level network and the energetically superior network, which is composed of the top 10 reactions ranked by *P*, *r* or − Δ_*r*_ *G*. Among three rating factors, under the same O_2_ and pH conditions, the overlap rates of links selected based on −Δ_*r*_ *G* were lower than those based on *P* and *r* (Figure 3a), suggesting that microbes do not always prefer reactions with lower Δ_*r*_ *G* as energy‐sourcing reactions. Reactions with lower Δ_*r*_ *G* often involve thermodynamically reactive N_2_H_4_ and N_2_H_5_^+^ as reactants. However, maintaining these reactions at a steady state can be challenging because N_2_H_4_ and N_2_H_5_^+^ are quickly depleted, significantly reducing the reaction rates and power generation at steady state. Consequently, microbial growth may not sustainably rely solely on these reactions. The ANAMMOX process is currently the only microbial process that utilises N_2_H_4_ as an intermediate (Figure 2c), facilitated by electron recycling within the cell (Strous et al. 2006). This suggests that biological utilisation of N_2_H_4_ or N_2_H_5_^+^ might fundamentally require elaborate mechanisms to maintain their concentrations.

For all rating factors, *D*_com,*ω*_ consistently surpasses *D*_enz,*ω*_ across all O_2_ and pH conditions. This suggests that the selective pressure from energetic favorability has acted at the community level rather than the enzyme level. This does not necessarily imply group selection since a single species can possess multiple enzymes. However, given the strong metabolic interdependencies in microbial communities, and the influence of these interdependencies on energy acquisition, it is possible that selective pressure is exerted on the overall metabolic processes of the community (Seto and Iwasa 2019; Zelezniak et al. 2015). The set of links *E*_*ω*_ at relatively higher O_2_ levels significantly overlapped with the community‐level links and were particularly involved in the complete or partial nitrification processes (cf. Figure 2c). The reduced overlap in low O_2_ environments likely overlooks the role of other elements that provide reducing power, as DNRA and denitrification processes utilise organic matter, ferrous ion, hydrogen gas, hydrogen sulfide and methane as electron donors (Brunet and Garcia‐Gil 1996; Di Capua et al. 2019; Ettwig et al. 2016; Haroon et al. 2013).

To investigate how the Gibbs energy driving force affects the similarity between the energetically superior network and community network, we analysed the response of the average *D*_com,*P*_ across various O_2_ and pH conditions, while varying both the reference reaction rate constant (*k*_def_) and the thermodynamic weight (*p*). As shown in Figure 3b, the average *D*_com,*P*_ increased with higher *p*, and this trend was independent of *k*_def_. In this model, the steady state of the system is primarily determined by the thermodynamic stability of nitrogen compounds at elevated *p* values (Figure 1). Our findings support the hypothesis that microbial nitrogen energy metabolism has evolved to effectively harness nitrogen redox reactions within ecosystems, where the composition of nitrogen compounds is initially determined abiotically by their thermodynamic potential.

## 4 Discussion

### 4.1 Reactions With Significant Contributions to Power Generation

**FIGURE 4:**
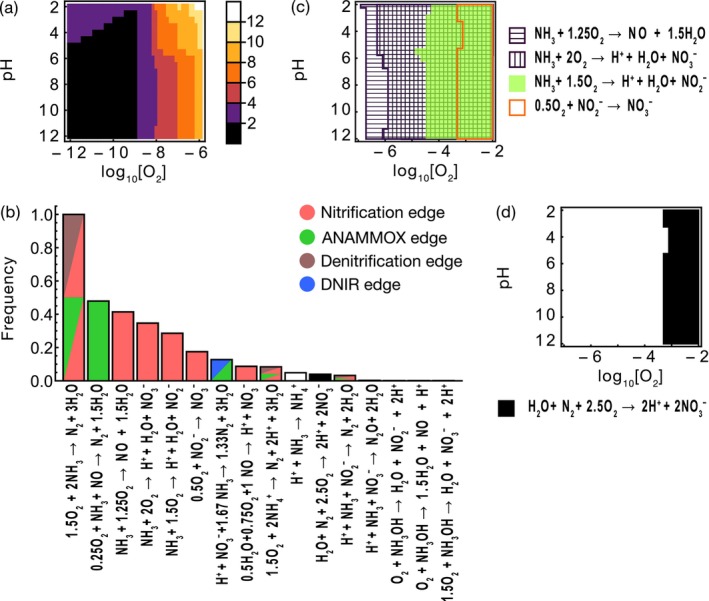
The set of reactions contributing to *θ* (0 ≤ *θ* ≤ 1) of the total power *P*_tot_. (a) The number of reactions *n* that represent 99.99% of the total power *P*_tot_ under 966 combinations of oxygen concentrations and pH conditions. (b) The frequency with which each key reaction appeared under 966 combinations of oxygen concentration and pH conditions. The colour of the bars indicates the relationship between the reactions and the corresponding enzyme function links; white and black denote reactions not included in enzyme links. (c) Conditions of oxygen concentration and pH under which complete ammonia oxidation and its partial reactions significantly contribute to the total power. (d) Oxygen concentration and pH conditions where the oxidation of N_2_ to NO_3_^−^ significantly contributes to the total power. *p* = 0.2, *k*_def_ = 1, *I* = 10^−5^, *D* = 10^−3^, *θ* = 0.9999 and *T* = 288.

We identified key reactions from the top‐ranked reactions that collectively contribute to the system's total power $P_{\mathrm{tot}}=\sum_{i=1}^{988}\max\left(0,P_{i}\right)$ by a fraction *θP*_tot_, where 0 ≤ *θ* ≤ 1. As *θ* approaches 1, the number of key reactions increases, as expected. However, a limited number of reactions dominate the contribution to the system's total power (Figure S3), even when *θ* = 0.9999 (Figure 4a). This is attributable to the strong thermodynamic constraints on energetically favourable reactions, particularly at higher *p*.

Figure 4b highlights key reactions across 966 O_2_ and pH conditions, indicating the frequency with which each reaction was identified as energetically superior. A composite reaction of nitrification and ANAMMOX, nitrification and denitrification, or DIRAMMOX was most frequently identified in both aerobic and anaerobic environments. Notably, among nitrification processes, the partial nitrification converting NH_3_ to NO was more frequently identified as a key reaction than complete ammonia oxidation, despite the Δ_*r*_ *G* of partial nitrifications generally being higher than that of complete ammonia oxidation. This prevalence is likely due to the lower oxygen demand per reaction, which allowed faster progression in low‐oxygen settings, thereby enhancing power generation (Figure 4c). Meanwhile, partial nitrifications that oxidise NH_3_ to NO_2_^−^ and NO_2_^−^ to NO_3_^−^ contributed significantly to the power only in environments with high oxygen concentrations, as their relatively rapid rates in low O_2_ environments do not offset the disadvantages of their higher Δ_*r*_ *G*.

### 4.2 Microbial Nitrogen Reaction Niches

Variations in the contributions of partial and complete nitrifications to power generation, based on O_2_ level in our calculations, may partially explain microbial niche specialisation. The relatively higher power generated by complete ammonia oxidation under low O_2_ conditions, as shown in our calculations, supports the preference of COMMAMOX *Nitrospira* for lower O_2_ conditions compared with partial nitrifiers (Dimitri Kits et al. 2017; Roots et al. 2019). However, at high O_2_ levels, the expected significant power from the partial nitrification converting NO_2_^−^ to NO_3_^−^ contrasts with reports of the inhibitory effect on the oxidation of NO_2_^−^ by certain nitrite‐oxidising bacteria (Sun et al. 2021). Partial and complete nitrifications utilising NH_3_ are identified as key reactions even in low pH environments, despite the increase in the NH_4_^+^/NH_3_ ratio as pH decreases. This may partially explain the prevalence of nitrifiers even under acidic conditions (pH < 5.5) (Li et al. 2018), while the use of urea as fertilisers could serve as an NH_3_ source in low pH soils in present‐day terrestrial ecosystems (Picone et al. 2021). It has been reported that ANAMMOX in marine environments can occur at O_2_ concentrations higher than the confirmed experimental O_2_ upper limit (approximately 20 μM) (Kalvelage et al. 2011). The higher power achievement by the combination of ANAMMOX and partial nitrification (reaction 3 in Figure 2b) suggests that ANAMMOX in such environments can be facilitated by the existence of an O_2_ sink due to the partial nitrification.

Among the key reactions, H^+^ + NH_3_ → NH_4_^+^ and H_2_O + N_2_ + 2.5O_2_ → 2H^+^ + 2NO_3_^−^ are excluded from the community‐level network. The latter has the potential to serve as an energy source for ATP synthesis. Since H_2_O + N_2_ + 2.5O_2_ → 2H^+^ + 2NO_3_^−^ contributes significantly to power generation only in high‐oxygen environments (Figure 4d), microbial enzymes catalysing this reaction, if discovered in the future, are likely to be found exclusively in such environments.

### 4.3 Partial Reaction Utilisation: Advantages and Diversity Insurance

Utilising partial reactions, despite their higher Δ_*r*_ *G*, can be energetically beneficial, exemplified by our model prediction. Similar to partial nitrifications, many denitrifiers rely on partial denitrification for ATP synthesis (Kuypers et al. 2018; Stein and Klotz 2016). Theoretical studies have shown that under specific conditions, partial reactions proceed more rapidly, thus enhancing ATP production (Costa et al. 2006; Pfeiffer et al. 2001; Pfeiffer and Bonhoeffer 2004). Moreover, integrating these reactions within microbial communities boosts overall energetic efficiency and power generation, surpassing the outcome of complete reactions alone (Seto and Kondoh 2023). The utilisation of partial reaction niches may also mitigate the competition for nitrogen sources by allowing them to differentiate niches. This diversity serves as an insurance effect (Yachi and Loreau 1999), ensuring the resilience and continuity of nitrogen transformation pathways within the nitrogen network.

## 5 Conclusions

This study tested the hypothesis that the energetic favorability of redox reactions, inherent in the thermodynamic potential of nitrogen compounds, shapes microbial nitrogen energy metabolism. By evaluating 988 theoretically plausible nitrogen reactions, we identified energetically superior reactions and compared them to energy‐sourcing reactions utilised by microbes. A graph‐theoretical analysis revealed strong agreement between these two sets of reactions, providing robust support for the hypothesis. Furthermore, our results showed that composite enzymatic reactions, rather than individual enzymatic reactions, tend to align more closely with energetically superior nitrogen reactions. This suggests that the selective pressure exerted by the energetic favorability of redox reactions appears to operate at the species or community level rather than the enzymatic level. These findings underscore the fundamental role of thermodynamics in shaping microbial metabolic networks at higher levels of biological organisation. Future research could explore how these perspectives and approaches extend to other energy metabolisms, such as those involving carbon and sulfur, contributing to a deeper understanding of the coevolution of ecosystem functions and microbial communities.

## Author Contributions

**Mayumi Seto:** conceptualization, methodology, investigation, validation, supervision, funding acquisition, writing – original draft, visualization, software, formal analysis, project administration. **Risa Sasaki:** validation, visualization, formal analysis, investigation, writing – original draft. **Hideshi Ooka:** conceptualization, methodology, data curation, funding acquisition, writing – review and editing. **Ryuhei Nakamura:** conceptualization, data curation, funding acquisition, writing – review and editing, project administration.

## Conflicts of Interest

The authors declare no conflicts of interest.

## Supporting information


Data S1.

## Data Availability

All codes were written in the Wolfram Language platform using Mathematica 12. All codes and Supplemental Information are archived in Dryad (https://doi.org/10.5061/dryad.3j9kd51ss) and Zenodo (https://doi.org/10.5281/zenodo.14768697).
