# Supplementary material for: Thermodynamics Underpinning the Microbial Community‐Level Nitrogen Energy Metabolism
Source: Environ Microbiol. 2025 Feb 16;27(2):e70055. doi: 10.1111/1462-2920.70055 (PMC11830459; doi:10.1111/1462-2920.70055)
Supplement: Supplementary file 1 — Data S1. [file EMI-27-e70055-s001.zip › SI/SI_Figure/SI_Figs.pdf]

Supplementary Figure 1

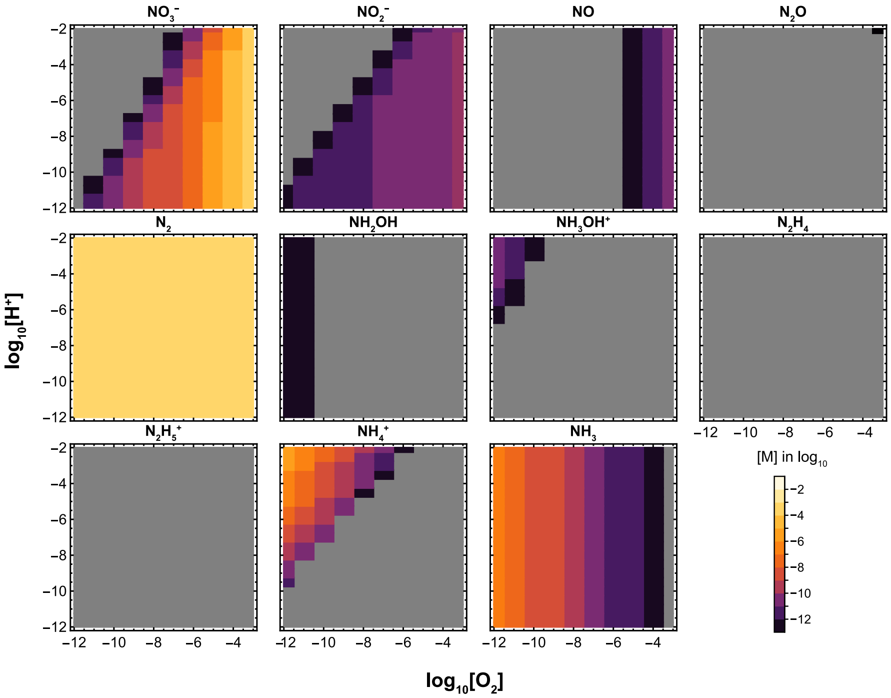

Figure 1: Steady-state concentrations of nitrogen species at various oxygen concentrations and pH levels. The gray areas indicate concentrations below 10^{-12} M. $p = 0.2$, $k_{def} = 10^0$, $I = 10^{-5}$, $D = 10^{-3}$, and $T = 288$K

Supplementary Figure 2

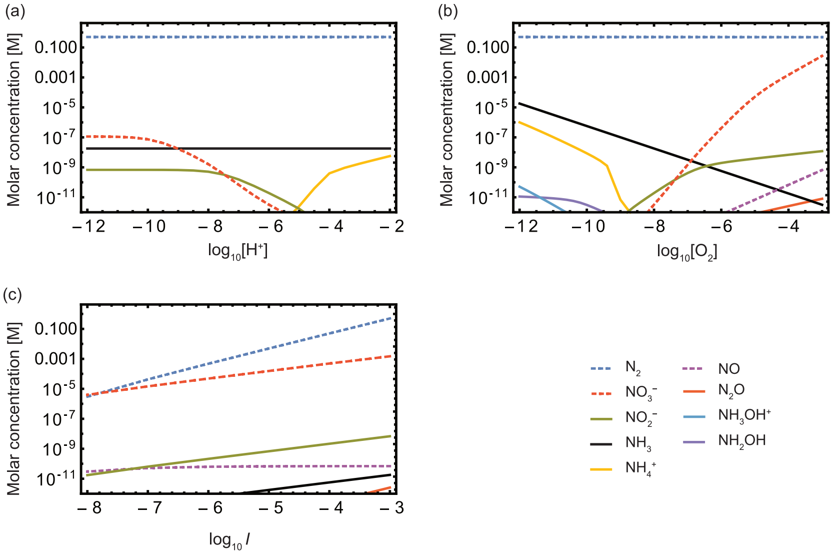

Figure 2: Steady state response of nitrogen species concentrations to variations in hydrogen ion concentration (a), oxygen concentration (b), and ammonia influx (c). (a) $[O_2] = 10^{-8}$ and $I = 10^{-3}$, (b) $[H^+] = 10^{-6}$ and $I = 10^{-3}$, (c) $[O_2] = 10^{-4}$ and $[H^+] = 10^{-6}$. Other parameter values are $p = 0.2$, $k_{\text{def}} = 10^0$, $D = 10^{-3}$, and $T = 288$.

Supplementary Figure 3

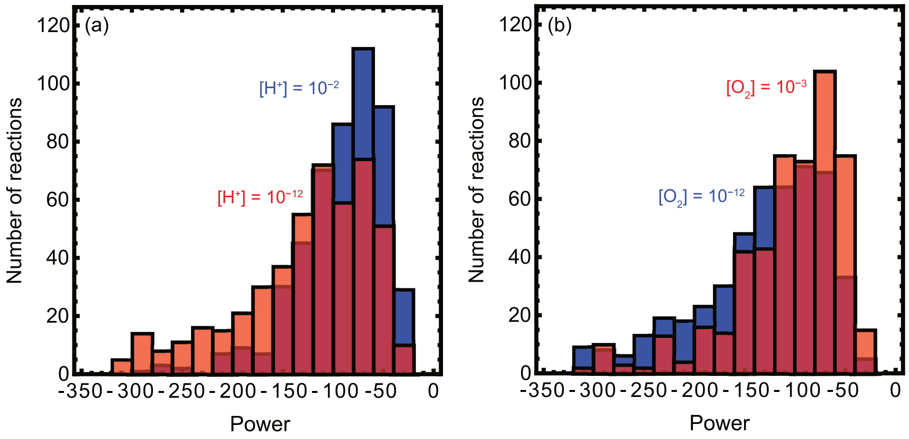

Figure 3: Distribution of the steady-state power generation $P_i = -\Delta_r G_i \times r_i$ for 988 reactions. (a) $[O_2] = 10^{-3}$ M, (b) pH = 8. Other parameter values are $I = 10^{-3}$, $p = 0.2$, $k_{\rm def} = 10^{-10}$, $D = 10^{-3}$, and $T = 288$ K.
