# Supplementary material for: Thermodynamics Underpinning the Microbial Community‐Level Nitrogen Energy Metabolism
Source: Environ Microbiol. 2025 Feb 16;27(2):e70055. doi: 10.1111/1462-2920.70055 (PMC11830459; doi:10.1111/1462-2920.70055)
Supplement: Supplementary file 1 — Data S1. [file EMI-27-e70055-s001.zip › SI/Supplementary_Table.doc]

**Supplementary Table:** Symbols and Abbreviations. For vectors and matrices, the specified unit represents the unit of their individual elements.

| Symbol /  Abbreviation | Unit | Description |
| --- | --- | --- |
| *rxn* | − | Reaction |
| **M**(*t*) | mol L^−1^ | Vector representing the molar concentrations of 11 nitrogen compounds: NO_3_^−^, NO_2_^−^, NO, N_2_O, N_2_, NH_2_OH, NH_3_OH^+^, N_2_H_4_, N_2_H_5_^+^, NH_4_^+^, NH_3_ |
| **I** | mol L^−1^ s^−1^ | Vector representing the influx rates of 11 nitrogen compounds |
| **C** | Dimensionless | Stoichiometric coefficient matrix (dimensions: 11 × 988), representing 11 nitrogen compounds and 988 reactions |
| **r**(*t*) | mol L^−1^ s^−1^ | Vector representing the reaction rates of 988 reactions |
| *n_k_*_,_*_i_* | Dimensionless | Stoichiometric coefficient of the *k*th nitrogen compound in *rxn_i_* |
| *r_i_* | mol L^−1^ s^−1^ | Reaction rate of *rxn_i_* |
| *D* | s^−1^ | Efflux rate constant, fixed at 10^−3^ |
| *k_i_* = *k_i_*(Δ*_r_G_i_*º) | Varies with reaction order † | Reaction rate constant for *rxn_i_*, which depends on Δ*_r_G_i_*º, the standard Gibbs energy change of *rxn_i_* |
| *k_def_* | Varies with reaction order † | Reference reaction rate constant |
| *p* | Dimensionless | Thermodynamic weighting coefficient |
| Δ*_r_G_i_*º | kJ mol^−1^ | Standard Gibbs energy change of *rxn_i_* |
| Δ*_r_G_i_* | kJ mol^−1^ | Gibbs energy change of *rxn_i_* |
| *T* | K | Absolute temperature, fixed at 288.15 |
| *R* | kJ mol^−1^ K^−1^ | Universal gas constant |
| *P_i_* | W | Power as energy output throughout the progress of *rxn_i_*: *P_i_*　= −∆*_r_G_i_* × *r_i_* |
| *G*(*V*, *E*) | − | Graph with *V* (11 nitrogen compounds as nodes) and *E* (directed links), representing transformations from 988 reactions |
| *G’*(*V_ω_*, *E_ω_*) | − | **Model-derived energetically-superior nitrogen network**: Subgraph with *V_ω_* (selected nodes) and *E_ω_* (selected links), based on *ω* = −Δ*_r_G_i_*, *r_i_*, or *P_i_* |
| *G’*(*V_enz_*, *E_enz_*) | − | **Enzyme-level nitrogen network**: Subgraph with *V_enz_* (nodes from 13 enzymatic reactions) and *E_enz_* (links from enzymatic reactions) |
| *G’*(*V_com_*, *E_com_*) | − | **Community-level nitrogen network**: Subgraph with *V_com_* (nodes from all enzymatic combinations) and *E_com_* (links from all possible enzymatic combinations) |
| *\|E\|* | Dimensionless | Number of links in *E* |
| *D_enz,ω_* | Dimensionless | Edge overlap between *E_ω_* and *E_enz_* |
| *D_com,ω_* | Dimensionless | Edge overlap between *E_ω_* and *E_com_* |

† The unit of *k_def_* depends on the reaction order and is determined to ensure that *r_i_* has consistent units of mol L^−1^ s^−1^.
